# GFAP and NfL as fluid biomarkers for clinical disease severity and disease progression in multiple system atrophy (MSA)

**DOI:** 10.1007/s00415-024-12647-z

**Published:** 2024-09-10

**Authors:** Sabrina Katzdobler, Georg Nübling, Martin Klietz, Urban M. Fietzek, Carla Palleis, Alexander M. Bernhardt, Florian Wegner, Meret Huber, Sophia Rogozinski, Luisa-Sophie Schneider, Eike Jakob Spruth, Aline Beyle, Ina R. Vogt, Moritz Brandt, Niels Hansen, Wenzel Glanz, Kathrin Brockmann, Annika Spottke, Daniel C. Hoffmann, Oliver Peters, Josef Priller, Jens Wiltfang, Emrah Düzel, Anja Schneider, Björn Falkenburger, Thomas Klockgether, Thomas Gasser, Brigitte Nuscher, Christian Haass, Günter Höglinger, Johannes Levin

**Affiliations:** 1 Department of Neurology, University Hospital, LMU Munich, Marchioninistr. 15, 81377 Munich, Germany; 2 Munich Cluster for Systems Neurology, SyNergy, Munich, Germany; 3 German Center for Neurodegenerative Diseases, DZNE, Munich, Germany; 4 Graduate School of Systemic Neurosciences (GSN), Munich, Germany; 5 Department of Neurology, Hanover Medical School, Hanover, Germany; 6 Department of psychiatry and neuroscience, Charité-Universitätsmedizin Berlin, Berlin, Germany; 7 German Center for Neurodegenerative Diseases (DZNE), Berlin, Germany; 8 Department of Psychiatry and Psychotherapy, Charité, Berlin, Germany; 9 Department of Neurology, University of Bonn, Bonn, Germany; 10 German Center for Neurodegenerative Diseases (DZNE), Bonn, Bonn, Germany; 11 German Center for Neurodegenerative Diseases (DZNE), Dresden, Germany; 12 Department of Neurology, Technische Universität Dresden, Dresden, Germany; 13 Department of Psychiatry and Psychotherapy, University Medical Center Goettingen, University of Goettingen, Goettingen, Germany; 14 German Center for Neurodegenerative Diseases (DZNE), Magdeburg, Germany; 15 Institute of Cognitive Neurology and Dementia Research, Otto-Von-Guericke University, Magdeburg, Germany; 16 Clinic for Neurology, Medical Faculty, University Hospital Magdeburg, Magdeburg, Germany; 17 German Center for Neurodegenerative Diseases (DZNE), Tübingen, Germany; 18 Department for Neurodegenerative Diseases, Hertie Institute for Clinical Brain Research, University of Tübingen, Tübingen, Germany; 19 Department of Psychiatry and Psychotherapy, School of Medicine and Health, Technical University of Munich, Munich, Germany; 20 Neuropsychiatry Unit and Laboratory of Molecular Psychiatry, Charité, Universitätsmedizin Berlin and DZNE, Berlin, Germany; 21 Centre for Clinical Brain Sciences, UK Dementia Research Institute at the University of Edinburgh, University of Edinburgh, Edinburgh, UK; 22 German Center for Neurodegenerative Diseases (DZNE), Goettingen, Germany; 23 Neurosciences and Signaling Group, Institute of Biomedicine (iBiMED), Department of Medical Sciences, University of Aveiro, Aveiro, Portugal; 24 Institute of Cognitive Neuroscience, University College London, London, UK; 25 Dept. of Neurodegenerative Disease and Geriatric Psychiatry/Psychiatry, University of Bonn Medical Center, Bonn, Germany; 26 Chair of Metabolic Biochemistry, Faculty of Medicine, Biomedical Center (BMC), LMU Munich, Munich, Germany; 27 Clinical Mass Spectrometry Center Munich, Munich, Germany

**Keywords:** Multiple system atrophy, Fluid biomarkers, Neurofilament light chain, Glial fibrillary acidic protein, Neuroinflammation

## Abstract

**Background:**

Multiple system atrophy (MSA), an atypical parkinsonian syndrome, is a rapidly progressive neurodegenerative disease with currently no established fluid biomarkers available. MSA is characterized by an oligodendroglial α-synucleinopathy, progressive neuronal cell loss and concomitant astrocytosis. Here, we investigate glial fibrillary acidic protein (GFAP) and neurofilament light chain (NfL) as fluid biomarkers for differential diagnosis, assessment of clinical disease severity and prediction of disease progression in MSA.

**Methods:**

GFAP and NfL levels were analyzed in plasma and CSF samples of 47 MSA patients as well as 24 Parkinson’s disease (PD) and 25 healthy controls (HC) as reference cohorts. In MSA, biomarker levels were correlated to baseline and longitudinal clinical disease severity (UMSARS scores).

**Results:**

In MSA, GFAP levels in CSF and plasma predicted baseline clinical disease severity as indicated by UMSARS scores, while NfL levels predicted clinical disease progression as indicated by longitudinal changes in UMSARS scores. Cross-sectionally, NfL levels in CSF and plasma were significantly elevated in MSA compared to both PD and HC. Receiver operating curves (ROC) indicated high diagnostic accuracy of NfL for distinguishing MSA from PD (CSF: AUC = 0.97, 95% CI 0.90–1.00; plasma: AUC = 0.90, 95% CI 0.81–1.00).

**Discussion:**

In MSA, GFAP shows promise as novel biomarker for assessing current clinical disease severity, while NfL might serve as biomarker for prediction of disease progression and differential diagnosis of MSA against PD.

**Supplementary Information:**

The online version contains supplementary material available at 10.1007/s00415-024-12647-z.

## Introduction

Multiple system atrophy (MSA) is a rare neurodegenerative disease, characterized histopathologically by oligodendroglial α-synucleinopathy with progressive neuronal cell loss and concomitant reactive astrogliosis [1–4]. Clinically, MSA shows a variable combination of autonomic dysfunction, parkinsonism and/or cerebellar symptoms [5]. Based on the predominant motor phenotype, patients are either classified as MSA-parkinsonian type (MSA-P) or MSA-cerebellar type (MSA-C) [6, 7]. Unlike Parkinson’s disease (PD), the second most common neurodegenerative disease with an underlying neuronal α-synucleinopathy [8], MSA is characterized by a rapid disease course and poor response to symptomatic treatment. Mean age of onset commonly is in the sixth decade of life with a limited life expectancy of 8–9 years after first symptom onset [9, 10]. Due to the high symptom overlap between MSA and PD especially in early disease stages, clinical differentiation between these two α-synucleinopathies often is challenging [11]. In the light of upcoming disease-modifying trials [12, 13], in vivo biomarkers for MSA-type α-synucleinopathy are urgently needed for early differential diagnosis as well as objective assessment of disease severity.

Given the extent of astrocytic involvement in MSA, investigating astroglial fluid biomarkers, such as Glial fibrillary acidic protein (GFAP), holds particular promise in MSA [14]. Higher expression levels of GFAP have been shown in post-mortem MSA brain tissue compared to PD and healthy controls (HC) [15], while first data also show a detectable upregulation of GFAP in CSF and plasma in multiple neurodegenerative diseases, including atypical parkinsonian syndromes [16]. Neurofilament light chain (NfL) has been identified as reliable fluid biomarker for neuroaxonal damage in neurodegenerative diseases [17], with first studies showing higher NfL levels in MSA compared to PD [18, 19].

Here, we investigate GFAP and NfL in CSF and blood samples in a multicentric cohort of MSA patients compared to PD and HC. By including longitudinal clinical data, we investigate GFAP and NfL as in vivo biomarkers for differential diagnosis, clinical disease severity and prediction of disease progression in MSA.

## Methods

### Patient collective

MSA patients were consecutively recruited at the Department of Neurology, LMU University Hospital, LMU Munich and the Department of Neurology, Hannover Medical School between 2018 and 2022. PD patients and HC were recruited at the LMU Hospital and within the DESCRIBE and DANCER study of the German Center for Neurodegenerative Diseases (DZNE e.V.).^1^

All MSA patients were diagnosed according to Gilman criteria which were the diagnostic criteria valid during the recruitment period [7]. PD patients were diagnosed according to the MDS clinical diagnostic criteria for PD [7, 20]. Written informed consent was obtained from all participants and the study was approved by the local ethics committees (Munich #21–0315, #20–0997; Hanover 8666_BO_K_2019). Clinical disease severity was assessed using the UMSARS I + II sum score for MSA (Unified Multiple System Atrophy Rating Scale) [21] and the MDS-UPDRS motor score (part III) for PD (MDS Unified Parkinson’s Disease Rating Scale) [22]. In MSA, clinical disease progression over a 1-year follow-up interval was calculated as follows:

$$UMSARS_{progression} \; = \;\frac{{UMSARS I + II _{score at follow up} {-} UMSARS I + II _{score at baseline}}}{{\Delta t \left( {date follow up - date baseline} \right) _{in years}}}$$

### Biosamples and analysis

Non-fasting EDTA plasma samples and serum samples, collected by venipuncture, and CSF samples, collected by lumbar puncture into polypropylene tubes, were centrifuged at 2000 × *g* for 10 min at room temperature and stored at – 80 °C until further analysis. All biosamples were analyzed in duplicates using the Quanterix Simoa^®^ Neurology 2-Plex B or 4-Plex B assay kit (Quanterix, Billerica, MA) according to the manufacturer’s instructions. In 13 MSA patients, only serum samples were available. Based on matching plasma and serum samples from the same study site, a coefficient to transform NfL and GFAP serum values into plasma values was calculated (see Supplement S1). Hence, in the following, “plasma” refers to both the values of analyzed plasma samples and the calculated “plasma” values of analyzed serum samples.

### Statistics

Statistical analyses were performed with SPSS Statistics 27.0 (IBM, Armonk, NY). Data were tested for normality using the Shapiro–Wilk test. For linear regression analysis, data were log-transformed if necessary to achieve normal distribution. Mann–Whitney *U* test and Kruskal–Wallis test were used for non-parametric group comparisons, while Spearman correlation was used for correlation analysis. Bonferroni–Correction was used to control for multiple testing. For diagnostic accuracy, receiver operating characteristic (ROC) curves were plotted and the area under the curve (AUC) was calculated. Optimal cutoff values were calculated using the Youden index [23]. A value of *p* < 0.05 was considered statistically significant.

## Results

Detailed clinical data and available biomaterial of both cohorts are provided in Table 1. A total of 47 MSA, 24 PD and 25 HC were recruited for this study. All groups were age matched, with the MSA and PD cohort demonstrating similar age of symptom onset and disease duration. Clinical follow-up data were available in 18 of 47 MSA patients.

**Table 1:** Demographical and biomarker data of all three cohorts included in this study

	MSA	PD	HC	*p* value
Total number (n)	47	24	25	
Sex (female vs. male)	16 vs. 31	12 vs. 12	11 vs. 14	*p* = 0.41
Age (y)	59.9 ± 7.8	59.7 ± 8.7	62.4 ± 9.2	*p* = 0.14
Disease duration (y)	4.0 ± 1.9	4.4 ± 3.3	–	*p* = 0.70
Age at first symptom onset	56.1 ± 9.1	55.3 ± 8.0	–	*p* = 0.91
MSA-C vs. MSA-P	25 vs. 22	–	–	
Probable vs. possible MSA^1^	32 vs. 15	–	–	
UMSARS I + II (baseline)	43.8 ± 13.9	–	–	
MDS-UPDRS III (baseline)	43.1 ± 17.8	25.1 ± 12.2	–	*p* < 0.001
Patients with available follow-up data (n)	18	–	–	
Change in UMSARS I + II over 12-month follow-up	11.3 ± 12.2	–	–	
**Biomarkers**				
Blood samples (n)	47	23	21	
Plasma/serum samples (n)	34/13^2^	23/0	21/0	
GFAP [pg/ml]	111.8 ± 61.6	93.9 ± 50.1	102.1 ± 78.2	*p* = 0.45
NfL [pg/ml]	24.3 ± 8.8	10.9 ± 9.0	11.4 ± 4.8	*p* < 0.0001^#.†^
CSF samples (n)	15	14	14	
GFAP [pg/ml]	17,904.7 ± 6704.3	13,885.0 ± 5074.4	9495.0 ± 5676.1	*p* < 0.01^#^
NfL [pg/ml]	4580.1 ± 2254.2	748.1 ± 350.8	703.4 ± 262.2	*p* < 0.0001^#.†^
CSF samples with matched plasma samples (n)	15	13	10	

Mean values ± SD. ^1^According to Gilman criteria. ^2^For serum samples, corresponding plasma levels were calculated using a transformation equation based on matched serum and plasma samples (see Supplement S1)

†MSA vs. PD

#MSA vs. HC

**Fig. 1:**
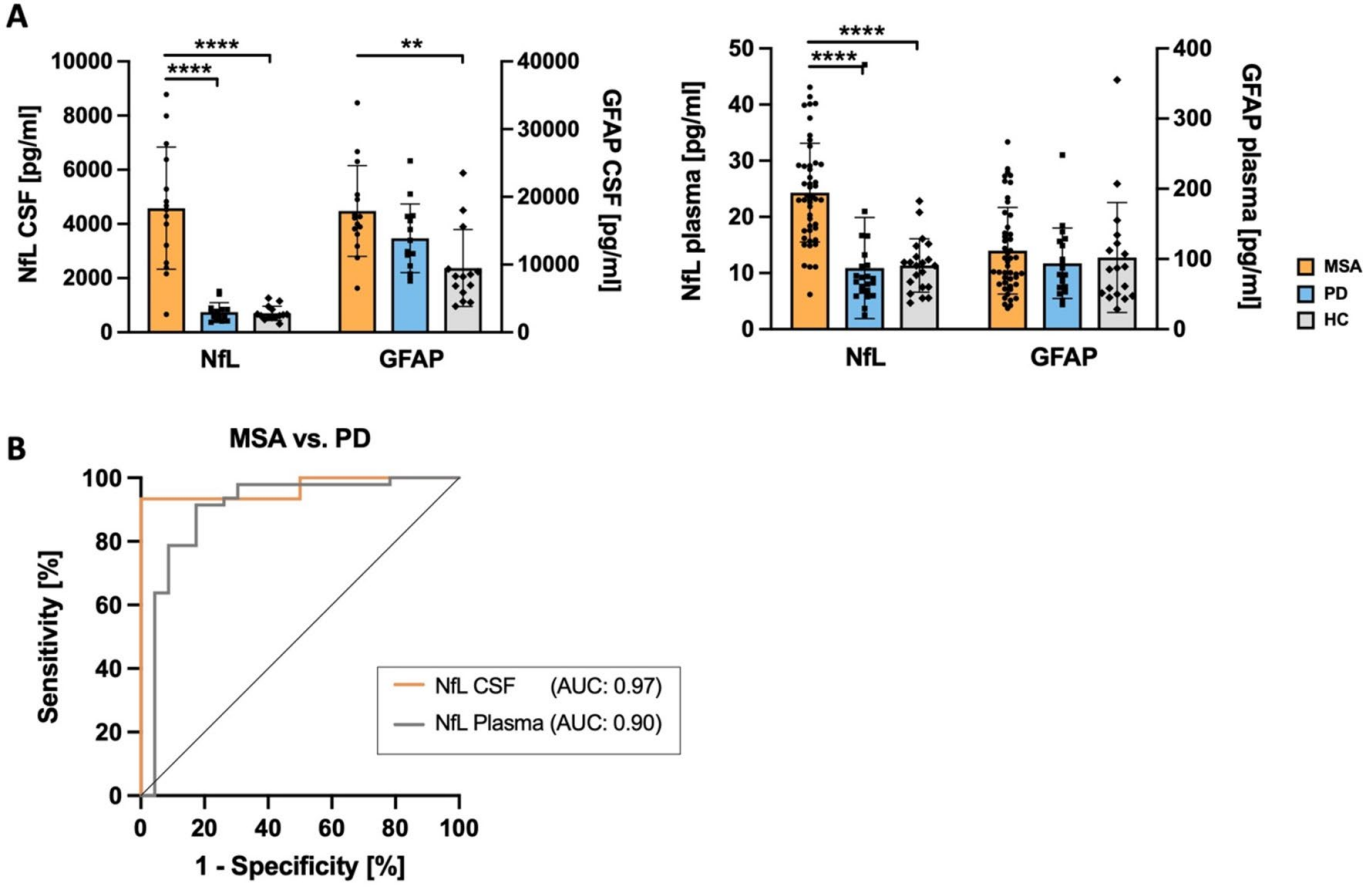
**NfL and GFAP as biomarkers in MSA, PD and HC.** **A** Biomarkers for differential diagnosis. NfL levels are significantly increased in both CSF and plasma of MSA compared to PD and HC. GFAP levels in CSF are significantly higher in MSA compared to HC. ****p < 0.0001. No significant changes in GFAP plasma levels were observed between the three groups. **B** Receiver operating characteristic (ROC) curve analysis for NfL in CSF and plasma show high AUC values for discrimination of MSA from PD patients (CSF: AUC = 0.97; plasma: AUC = 0.90)

In both CSF and plasma, NfL was significantly elevated in MSA compared to PD and HC (*p* < 0.001), while no significant difference was found for NfL between PD and HC. In CSF, GFAP was significantly elevated in MSA compared to HC (*p* < 0.01), while no difference was found between MSA and PD as well as PD and HC. GFAP levels in plasma did not differ between groups (Fig. 1A). In MSA, no significant differences between MSA-C and MSA-P subgroups were found for either biomarker in CSF and plasma.

To further assess the diagnostic accuracy of NfL for discrimination of MSA from PD, ROC analyses were performed (Fig. 1B). AUC values indicated high diagnostic accuracy for NfL in CSF (AUC = 0.97, *p* < 0.0001, 95% CI 0.90–1.00) with only slightly lower diagnostic accuracy in plasma (AUC = 0.90, *p* < 0.0001, 95% CI 0.81–1.00). For NfL, in CSF, an optimal cutoff value of 1835.0 pg/ml and in plasma, an optimal cutoff value of 14.07 pg/ml was determined using the Youden index.

**Fig. 2:**
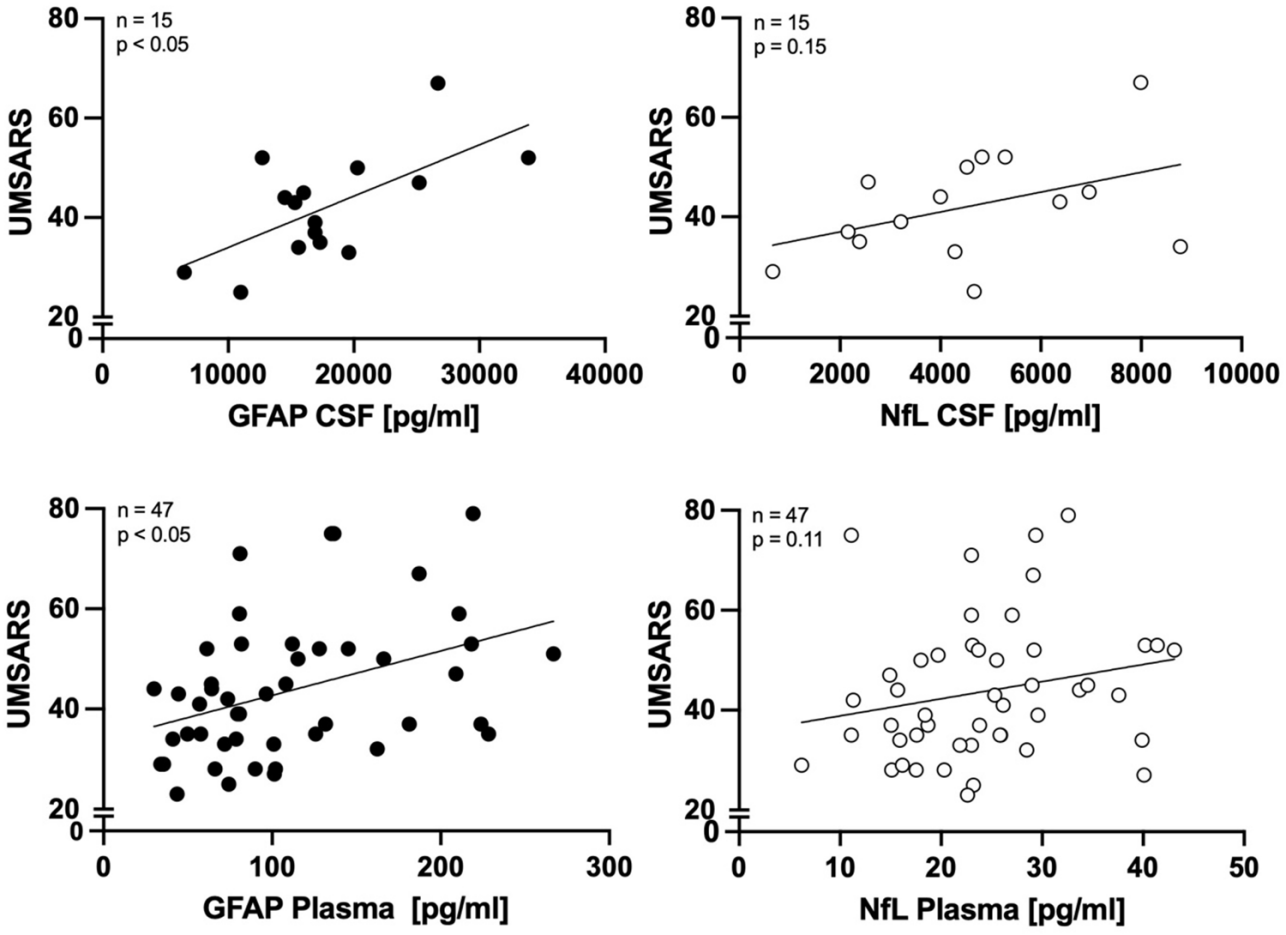
**Biomarkers for clinical disease severity in MSA.** CSF and plasma levels of GFAP and NfL in MSA compared to UMSARS I + II scores. Multiple regression analysis adjusting for age-identified GFAP levels in CSF and plasma as significant predictor of baseline UMSARS I + II scores in MSA patients, but not NfL

To investigate NfL and GFAP as biomarkers for disease severity in MSA, multiple linear regression analysis was performed to predict UMSARS scores using either GFAP or NfL as biomarker in CSF or plasma, respectively, adjusting for age as confounder. The overall regression model using GFAP as biomarker was statistically significant for both CSF and plasma levels (CSF GFAP: F(2, 11) = 4.012, *p* < 0.05; plasma GFAP: F(2, 44) = 5.246, *p* < 0.01) with an R squared of 0.424 and 0.193, respectively. In each model, GFAP levels were significant predictors of baseline UMSARS scores assessed at the time point of biomaterial sampling (CSF: *β* = 0.678, *p* =  < 0.05; plasma: *β* = 0.354, *p* < 0.05), while age did not significantly predict UMSARS scores within the model. Regression analysis did not support NfL in either CSF or plasma as significant predictor for baseline UMSARS scores assessed at the time point of sampling in MSA (Fig. 2).

**Fig. 3:**
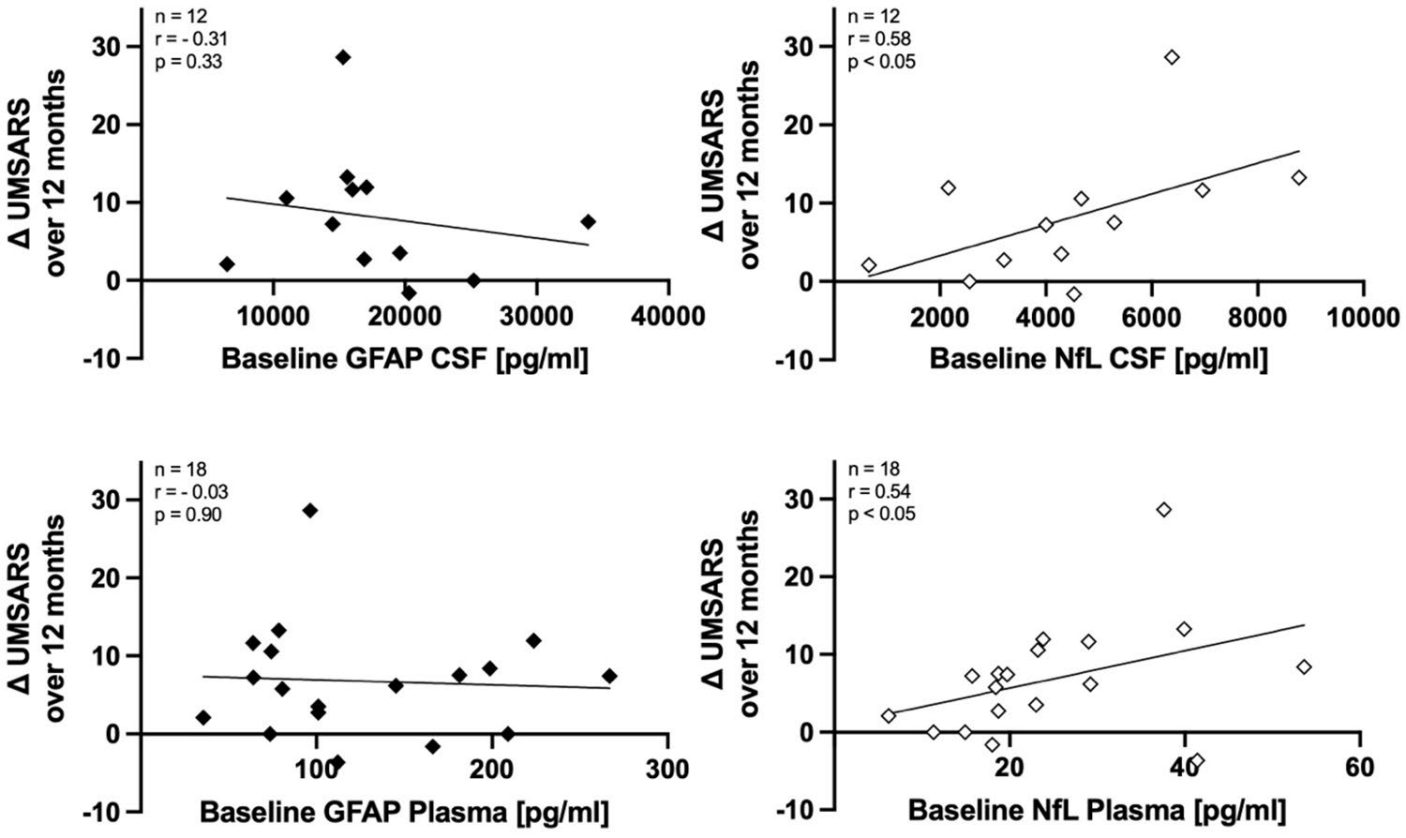
**Biomarkers for prediction of disease progression in MSA.** While no correlation was found for baseline levels of GFAP with longitudinal change in UMSARS scores over 12 months, CSF and plasma levels of NfL show significant positive correlation with longitudinal change in UMSARS scores over 12 months in MSA patients, indicating prediction of disease progression

To investigate NfL and GFAP as biomarkers for prediction of clinical disease progression, baseline levels of both biomarkers in CSF and plasma were correlated to longitudinal change in UMSARS scores calculated over a 1-year follow-up period. For NfL, significant positive correlation with UMSARS_progression_ values was found for CSF (*r* = 0.64, *p* < 0.05) and plasma (*r* = 0.54, *p* < 0.05), while no correlation was found for GFAP in either CSF or plasma (Fig. 3).

## Discussion

Our results indicate that reactive astrogliosis measured by GFAP is associated with clinical disease severity in MSA, while neuronal cell loss assessed by NfL predicts progression of clinical symptoms. On a biomarker level, GFAP might be a suitable biomarker in MSA for assessment of current clinical disease severity, while NfL might serve as biomarker for prediction of clinical disease progression.

The temporal relationship between reactive astrogliosis and MSA-type α-synuclein pathology throughout the disease course still remains elusive with limited in vivo biomarker studies available in MSA [24]. In neuropathological investigations, reactive astrogliosis has been reported to parallel distribution of α-synucleinopathy in MSA [25]. In vitro studies suggest direct activation of astrocytes by α-synuclein itself [26, 27].

To the best of our knowledge, this is the first study to investigate the relationship between reactive astrogliosis and clinical disease severity in a cohort of MSA patients in vivo using GFAP as surrogate biomarker in a longitudinal clinical dataset. Our findings indicate that reactive astrogliosis reflects clinical disease severity in MSA patients and thereby potentially severity of disease pathology itself. Reactive astrogliosis is most likely a dynamic process in other neurodegenerative diseases such as Alzheimer’s disease (AD) or PD, indicating its broader role in neurodegeneration. Interestingly, an increase in plasma GFAP levels despite decreasing PET-signals for astrogliosis over time was seen in AD [28], while an in vivo PET-imaging study in PD showed an initial upregulation followed by a downregulation of reactive astrogliosis over the disease course [29]. Further investigations of GFAP and other biomarkers targeting astrogliosis will be needed to better understand the dynamic of reactive astrogliosis throughout the disease course of MSA and its implication for biomarkers of disease severity.

Regarding cross-sectional differences in GFAP in levels between disease groups, we found overall higher CSF GFAP levels in MSA compared to HC, but not PD. This is in line with another recent in vivo cross-sectional study also using an ultra-sensitive immunoassay for detection of GFAP in CSF [16], while earlier in vivo studies were not able to detect differences in CSF GFAP levels between MSA and HC [30, 31], possibly due to limited assay sensitivity.

NfL has long been established as biomarker for neuroaxonal damage. Corroborating earlier findings [18, 19], our study found significantly elevated NfL levels in MSA compared to PD and HC. This is well in line with the more aggressive disease course and pronounced cell loss in MSA pathology compared to PD. Reflecting this difference in disease severity, NfL shows potential to facilitate differential diagnosis between patients with MSA pathology and PD pathology (“diagnostic biomarker”) [32]. In addition, with NfL indicating neuronal cell loss, it is well matching, that higher NfL levels predict higher increases in UMSARS scores over time, i.e., progression of neurological symptoms. However, it is important to acknowledge that NfL is not specific to the underlying pathology. Other studies have reported elevated NfL levels in patients with more aggressive neurodegenerative disease entities such as progressive supranuclear palsy (a four-repeat-(4R)-tauopathy), corticobasal syndrome (distinct underlying histopathologies, including 4R-tauopathies and mixed 3/4R tauopathy of AD pathology among others) or amyotrophic lateral sclerosis [16, 33]. In the spectrum of synucleinopathies, besides in MSA, elevated Nfl levels have also been observed in PD patients with rapid disease progression when compared to slowly progressing PD patients [16, 33, 34]. Elevated NfL levels were also reported in PD patients in the months following DBS surgery [35], showing the limited specificity of NfL regarding the cause of neuroaxonal damage.

Nevertheless, when carefully evaluating and excluding potential confounders of elevated NfL levels, i.e., brain surgery, trauma or stroke, our study supports NfL as biomarker to identify patients with an underlying rapidly progressive neuropathology, such as MSA. Other potential biomarkers for the differential diagnosis of MSA from PD and other atypical parkinsonian syndromes include MIBG scintigraphy for assessment of cardiac sympathetic denervation, MRI imaging for atrophy patterns and with more recent advancements the investigation of skin biopsies to detect alpha-synuclein deposits and novel seeding aggregation assays for α-synuclein in CSF [6, 36, 37]. Until biomarkers that provide definitive in vivo proof of MSA pathology, such as seeding assays and skin biopsies, become reliable and widely accessible, biomarkers like NfL in combination with other biomarkers such as MRI imaging and MIBG scintigraphy may be utilized to facilitate the differential diagnosis of MSA from PD.

It must be noted that in our cohort, only a trend towards a positive association of CSF and plasma NfL levels with baseline UMSARS scores was observed, whereas previous studies reported such associations to be significant [38, 39]. One reason might be the smaller cohort size in this study when compared to these other studies. Nonetheless, this did not hinder the identification of GFAP as potential biomarker for clinical disease severity in our cohort. Considering MSA being a rare disease, clinically relevant biomarkers must not only be detectable in large cohorts but should also be valid in smaller sample sizes.

One general limitation of this study shared with most in vivo biomarker studies in MSA is the lack of neuropathological confirmation of diagnosis. To minimize the risk of clinical misdiagnosis, all patients included in this study have been seen in specialized departments for movement disorders and two-thirds of MSA patients fulfilled diagnostic criteria for “probable MSA” according to Gilman criteria. It must be noted, however, that in 2022, the new Movement Disorder Society criteria for the diagnosis of MSA have been published [6]. These new criteria were published to increase sensitivity and specificity regarding the clinical diagnosis of MSA patients especially in early disease stages. In multiple recent retrospective post-mortem validation studies, these new diagnostic criteria have shown excellent specificity, however with low to moderate sensitivity for the diagnosis of MSA across different disease stages [40–42]. Mixed results have been reported regarding their performance against the Gilman criteria, with most studies agreeing on higher, yet still limited sensitivity of the new MDS MSA criteria with similar or slightly overall increased specificity. Since these new criteria were released after the recruitment period of this study, and considering that the latest validation study suggests the best sensitivity combined with high specificity for trial selection using the Gilman criteria categories (possible and probable MSA) or the MDS MSA criteria (clinically probable and clinically established MSA) [40], we opted to continue using the Gilman criteria for this study.

In summary, our study is the first to show to the potential of GFAP as objectively measurable fluid in vivo biomarker for clinical disease severity in MSA. Such biomarkers are currently urgently needed for clinical trials. In addition, it supports previous studies on NfL as fluid in vivo biomarker in MSA for prediction of disease progression as well as facilitation of differential diagnosis against PD, which currently often still poses a clinical challenge. Our findings warrant follow-up investigations in a larger, longitudinal MSA cohort to address the dynamics of reactive astrogliosis and neuronal cell loss during MSA and PD pathology and to further establish GFAP and NfL as in vivo biomarkers for MSA.

## Supplementary Information

Below is the link to the electronic supplementary material.

Supplementary file1 (PPTX 95 KB)

## Data Availability

The data presented in this study are available on reasonable request and as patient consent allows from the corresponding author.

## Footnotes

1 The DESCRIBE study (DZNE Clinical Register Study of Neurodegenerative Disorders) is a German wide prospective multicenter observational cohort study, recruiting patients with various neurodegenerative diseases, including PD and atypical parkinsonian syndromes such as MSA. Patients are consecutively enrolled at one of the 11 participating tertiary care centers with clinical follow-up visits every 12 months. DANCER (Degeneration Controls and Relatives) is a parallel prospective multicenter observational cohort study recruiting healthy controls within the same DZNE research network.
