# Supplementary material for: GFAP and NfL as fluid biomarkers for clinical disease severity and disease progression in multiple system atrophy (MSA)
Source: J Neurol. 2024 Sep 10;271(10):6991–9. doi: 10.1007/s00415-024-12647-z (PMC11447157; doi:10.1007/s00415-024-12647-z)
Supplement: Supplementary file 1 — Supplementary file1 (PPTX 95 KB) [file 415_2024_12647_MOESM1_ESM.pptx]

## Slide 1
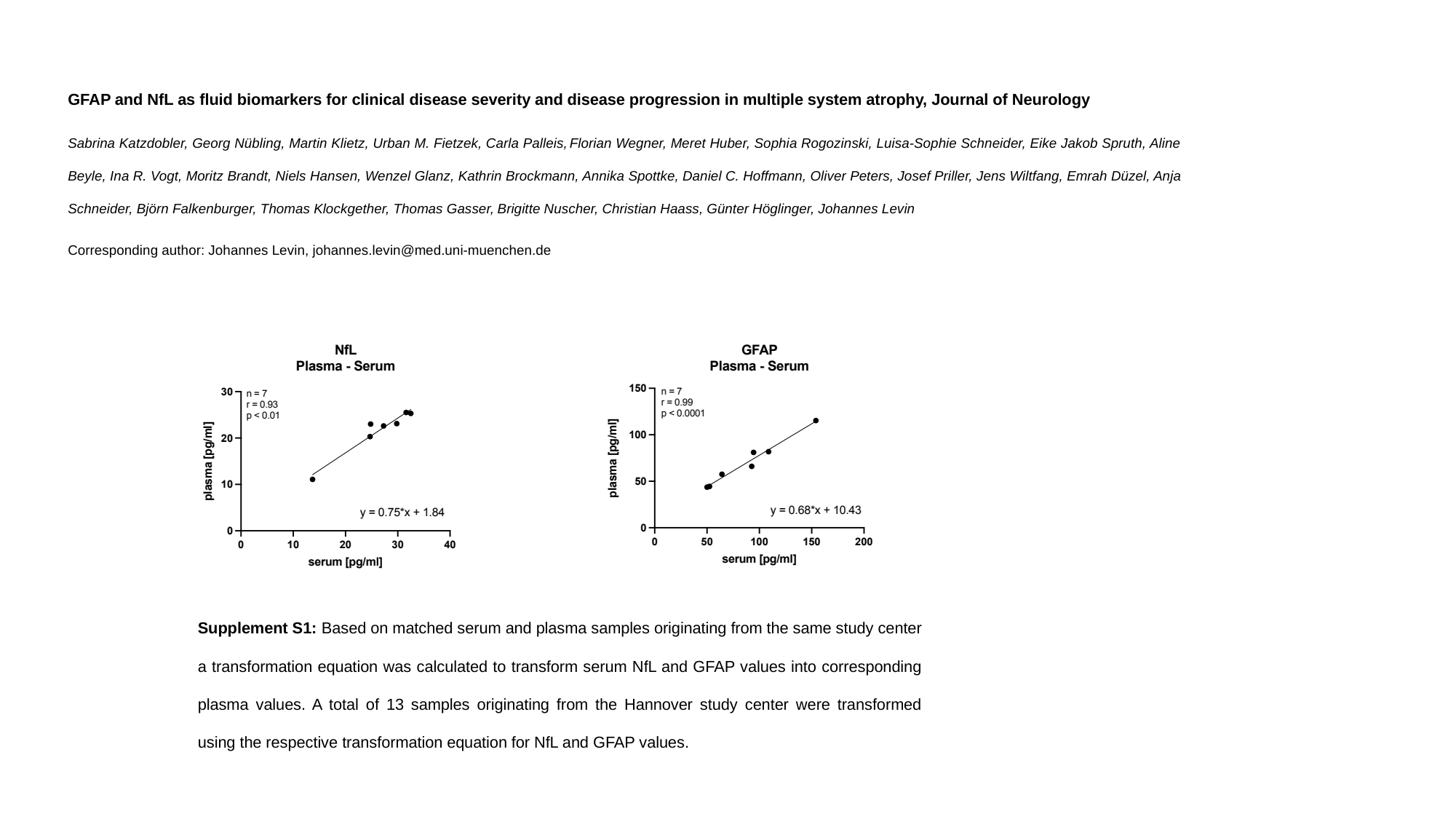

GFAP and NfL as fluid biomarkers for clinical disease severity and disease progression in multiple system atrophy, Journal of Neurology
Sabrina Katzdobler, Georg Nübling, Martin Klietz, Urban M. Fietzek, Carla Palleis, Florian Wegner, Meret Huber, Sophia Rogozinski, Luisa-Sophie Schneider, Eike Jakob Spruth, Aline Beyle, Ina R. Vogt, Moritz Brandt, Niels Hansen, Wenzel Glanz, Kathrin Brockmann, Annika Spottke, Daniel C. Hoffmann, Oliver Peters, Josef Priller, Jens Wiltfang, Emrah Düzel, Anja Schneider, Björn Falkenburger, Thomas Klockgether, Thomas Gasser, Brigitte Nuscher, Christian Haass, Günter Höglinger, Johannes Levin
Corresponding author: Johannes Levin, johannes.levin@med.uni-muenchen.de
Supplement S1: Based on matched serum and plasma samples originating from the same study center a transformation equation was calculated to transform serum NfL and GFAP values into corresponding plasma values. A total of 13 samples originating from the Hannover study center were transformed using the respective transformation equation for NfL and GFAP values.
